# Diverticulitis With Microperforation

**DOI:** 10.7759/cureus.27159

**Published:** 2022-07-22

**Authors:** Rhea Choksey, Thor S Stead, Rohan Mangal, John Amatea, Latha Ganti

**Affiliations:** 1 Biology, Trinity Preparatory School, Winter Park, USA; 2 Medicine, The Warren Alpert Medical School of Brown University, Providence, USA; 3 Medicine, University of Miami Miller School of Medicine, Miami, USA; 4 Emergency Medicine, Lakeland Regional Health, Lakeland, USA; 5 Emergency Medicine, HCA Florida Ocala Hospital, Ocala, USA; 6 Emergency Medicine, Envision Physician Services, Plantation, USA; 7 Emergency Medicine, University of Central Florida College of Medicine, Orlando, USA

**Keywords:** sigmoid diverticulitis, perforated diverticulitis, microperforation, complicated diverticulitis, diverticulitis recommendations

## Abstract

The authors present the case of a 39-year-old male who returned to the emergency department one month after uncomplicated diverticulitis, with the second bout of diverticulitis newly complicated by microperforation. The clinical presentation, diagnosis, and management of acute diverticulitis across the spectrum of presentations are discussed.

## Introduction

Diverticulitis is inflammation or infection of the diverticula. Diverticula can form when weak spots in the colon give way to pressure, which causes sac-like protrusions through the muscular layer (which does not contain all the layers of the bowel wall), referred to as diverticulosis. The lifetime prevalence of developing acute diverticulitis in persons with diverticulosis is approximately 25% [1]. Patients with diverticulitis usually present with fever, pain, specifically in the lower left abdomen (depending on which side of the colon is affected, in the Asian population right side is also common), nausea, vomiting, abdominal tenderness, and constipation.

Acute diverticulitis accounts for more than 2.6 million outpatient visits and 200,000 inpatient admissions each year in the United States [2]. Overall, the incidence of diverticulitis is increasing. A query of the National Emergency Department Sample showed acute diverticulitis-related emergency department (ED) visits increased by 27% between 2006 and 2013 [3]. There are several risk factors for acute diverticulitis. Obesity, defined as a basal metabolic index (BMI) of >30 kg/m^2^ is a risk factor that is on the rise as parts of the world are steadily becoming more obese [4]. This risk can be related to the overall weight or to the percent of visceral fat, both of which increase with obesity [5]. Other risk factors include aspirin, nonsteroidal anti-inflammatory drugs, lack of exercise, and increasing age [6]. Diverticulitis risk can be decreased by exercising regularly, having a balanced diet with more fiber, drinking fluids, maintaining a healthy weight, and avoiding smoking [7].

## Case presentation

The patient is a 39-year-old male with no significant past medical history who presented to the ED with five days of left lower quadrant abdominal pain. The pain was aggravated by walking. The patient was seen one month prior and found to have uncomplicated diverticulitis. He was treated as an outpatient with one week course of cefdinir and metronidazole. The patient reported at this time that he also adjusted his diet and quit smoking. After taking the antibiotics his symptoms improved. However, approximately five days before his second visit his left lower quadrant pain returned. The patient reported subjective fevers, chills, and decreased stool content. He denied chest pain or shortness of breath. He denied any nausea or vomiting, diarrhea, or blood in his stool. The patient passed gas normally and his last bowel movement was the morning of his second visit.

Vitals signs in the ED revealed a temperature of 36.4°C, pulse rate of 106, respiration rate of 18, blood pressure of 137/80 mmHg, and oxygen saturation at 100% on room air. Physical exam demonstrated a non-peritonitic abdomen. He did have tenderness of the left lower quadrant and some mild rebound tenderness.

His laboratory analysis was significant for marked leukocytosis (Table 1).

**Table 1:** Patient’s laboratory analyses

Chemistry	Normal range	Results
Sodium	136-145 mmol/L	136
Potassium	3.7-5.1 mmol/l	3.6 L
Chloride	98-107 mmol/L	104
Carbon dioxide	21-32 mmol/L	22
Anion gap		13.6
Blood urea nitrogen (BUN)	7-18 mg/dL	10
Creatinine	0.55-1.3 mg/dL	1.09
Glucose	74-106 mg/dL	136 H
Calcium	8.4-10.1 mg/dL	9
Total bilirubin	0.2-1.5 mg/dL	0.6
Aspartate aminotransferase (AST)	10-37 unit/L	27
Alanine transaminase (ALT)	12-78 unit/L	58
Total alkaline phosphatase	45-117 unit/L	138 H
Total protein	6.4-8.2 g/dL	8.4 H
Albumin	3.4-5.0 g/dL	3.0 L
Lipase	0-160 unit/L	47
Hematology	Normal range	Results
White blood cells (WBC)	4.0-10.5 10^3/µL	22.3 H
Red blood cells (RBC)	4.63-6.08 10^6/µL	4.02 L
Hemoglobin (Hgb)	13.7-17.5 g/dL	12.1 L
Hematocrit (Hct)	40.1-51.0%	35.9 L
Mean corpuscular volume (MCV)	79.0-92.2 fL	89.3
Mean corpuscular hemoglobin (MCH)	25.7-32.2 pg	30.1
Mean corpuscular hemoglobin concentration (MCHC)	32.3-36.5 g/dL	33.7
Red cell distribution width (RDW)	11.6-14.1%	12.4
Pit count	150-400 10^3/µL	442 H
Mean platelet volume (MPV)	9.4-12.4 fL	9.7
Immature Gran %	0.0-0.4%	0.5 H
Neutrophils %	34.0-67.9%	76.1 H
Lymphocytes	21.8-53.1%	13.1 L
Monocytes %	5.3-12.2%	10
Eosinophils %	0.8-7.0%	0.1 L
Basophils %	0.1-1.2%	0.2
Nucleated RBC %	0.0-0.2%	0
Immature granulocytes	0.00-0.04 10^3/µL	0.11 H
Urinalysis	Normal	

**Figure 1:**
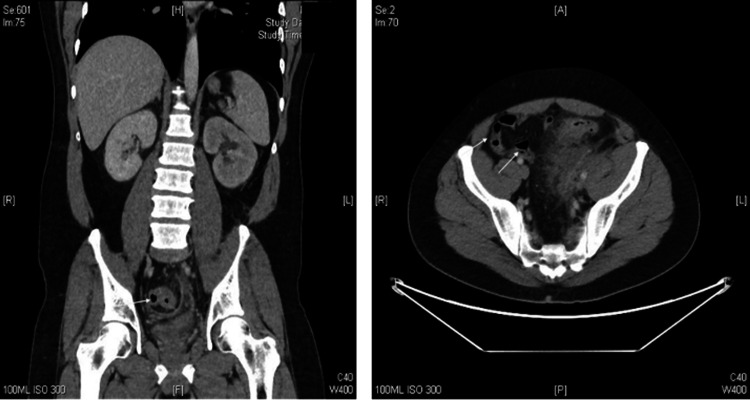
Computed tomography scan (coronal and axial planes) demonstrating microperforation (arrows)

Computed tomography (CT) scan of the abdomen and pelvis with contrast showed sigmoid diverticulitis with a small amount of gas and fluid noted in the surrounding mesocolon, consistent with microperforation. No drainable abscess collection was identified. Mild left hydronephrosis was noted, likely secondary to ureteral compression in the pelvis (Figure 1).

General surgery was consulted and they opined that there was no indication for surgical intervention at the time. The patient was given nothing by mouth for bowel rest and was admitted to the hospital for serial abdominal exams, analgesia, and continuation of intravenous antibiotics. The patient was discharged home three days later on oral antibiotics.

## Discussion

This case highlights that there is a spectrum of diverticular disease, from uncomplicated diverticulitis which the patient had on his initial presentation, to diverticulitis with microperforation, which the patient experienced on his second visit. Fortunately, he did not go on to have a gross perforation which would be even more severe.

Traditionally, uncomplicated diverticulitis has been treated with bowel rest and antibiotics, a practice reflected in a majority of surveyed colorectal surgeons [8,9]. However, the Efficacy and Safety of Nonantibiotic Outpatient Treatment in Mild Acute Diverticulitis (DINAMO study) randomized 480 patients to either classic treatment with antibiotics, or to treatment with anti-inflammatory medication. They found outpatient supportive, symptomatic treatment of mild acute diverticulitis without antibiotics to be safe, effective, and non-inferior to current standard treatment [10]. This trial and other studies have led to the 2022 practice-changing update to not routinely prescribe antibiotics for uncomplicated mild acute diverticulitis. A 2022 Cochrane Review confirms that this is no short-term benefit of antibiotics, based on the outcomes of 1329 patients in three studies [11].

Micro-perforations, as the name suggests, are very small perforations that show up as small air bubbles on the CT. Most cases of diverticulitis with microperforation can be managed with intravenous antibiotics as was our patient, and avoid operative intervention.

**Figure 2:**
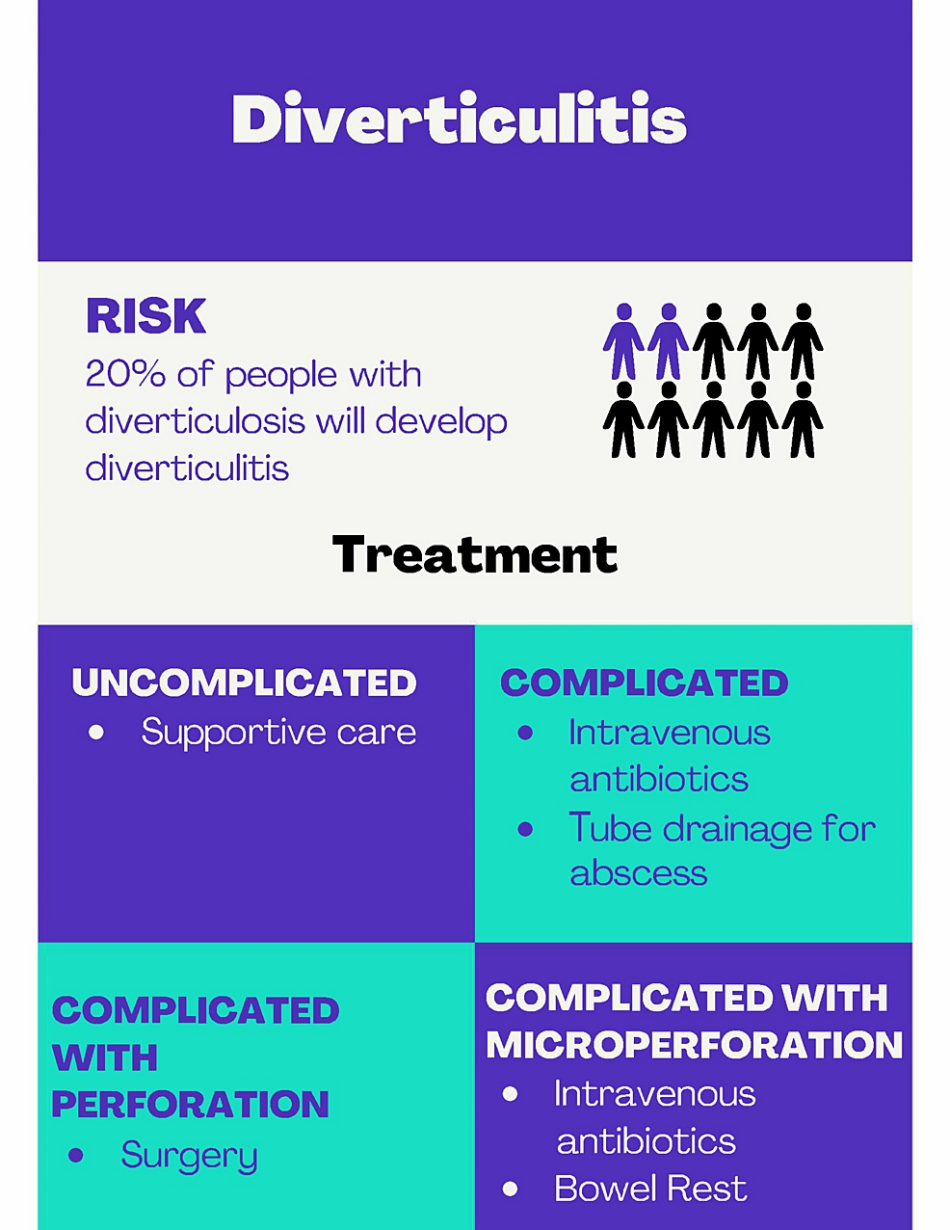
Infographic summarizing diverticulitis. Designed by Rhea Choksey on canva.com

Surgical intervention is necessary if diverticulitis is complicated by gross perforation. Perforation can be caused by trauma, inflammation, infection, or ischemia. Other complications of diverticulitis that may require surgical intervention include bowel abscess, fistulation, or obstruction. The type of operative intervention for diverticulitis with perforation depends on the patient’s hemodynamic status and the extent of the disease. These can include primary resection and anastomosis, Hartmann’s procedure, or damage control surgery (Figure 2).

The Hinchey classification for acute diverticulitis can be helpful in correlating clinical presentation with CT findings and potential management (Table 2).

**Table 2:** Hinchey Classification for acute diverticulitis

Stage	Clinical	CT findings	Management	
0	Mild clinical diverticulitis	Diverticula with colonic wall thickening	Observation, diet changes	
				Ia	Confined pericolic inflammation or phlegmon	Pericolic soft tissue changes	Antibiotics		
Ib	Pericolic or mesocolic abscess	Ia changes and pericolic or mesocolic abscess	Drainage by interventional radiology						
				II	Pelvic, distant intra-abdominal, or retroperitoneal abscess	Ia changes and distant abscess, usually deep pelvic	Drainage by interventional radiology		
III	Generalized purulent peritonitis	Localized or generalized ascites, pneumoperitoneum, peritoneal thickening	Surgical intervention						
				IV	Generalized fecal peritonitis	Same as stage III	Surgical intervention		

The “An Acute Care Surgery in the Netherlands” (ACCSENT trial) multicenter retrospective cohort study reported an 85% improvement rate in 101 patients with Hinchey Ia diverticulitis when treated with observation alone, antibiotics, and/or hospital admission [12]. Sixteen to 40% of diverticulitis cases are complicated by the presence of an abscess [13]. A diverticular abscess puts someone in the Hinchey Ib or II category, and these abscesses can be successfully managed with percutaneous catheter drainage [7]. The larger the abscess, the easier the percutaneous drainage [14]. Hinchey stages III and IV are managed operatively.

There is also a risk of recurrent diverticulitis, just like with this patient. Approximately 20% of patients with uncomplicated diverticulitis have a recurrence. After the second episode of diverticulitis, there is an 18% risk after one year of recovery for another recurrence, 55% risk after 10 years of recovery, and after a third episode, there is a 40% risk after 3 years of recovery. The risk of perforation or other complications after a recurrence is less than 6% [15].

## Conclusions

Diverticulitis is a common ED presentation. Recent guidelines from the American Gastroenterological Association suggest that antibiotics are no longer indicated for uncomplicated diverticulitis, which is a change in practice from previous. However, the presence of complicating factors alters management.
